# Experimental Efficacy of an Alphavirus Vectored RNA Particle Vaccine Against Porcine Parainfluenza Virus-1 in Conventional Weaned Pigs

**DOI:** 10.3390/v17040565

**Published:** 2025-04-14

**Authors:** Michael Welch, Karen Krueger, Jianqiang Zhang, Pablo Piñeyro, Mark Mogler, Erin Strait, Phillip Gauger

**Affiliations:** 1 Department of Veterinary Diagnostic and Production Animal Medicine, College of Veterinary Medicine, Iowa State University, 1800 Christensen Drive, Ames, IA 50011, USA; mwelch@hogvet.com (M.W.); kharmon@iastate.edu (K.K.); jqzhang@iastate.edu (J.Z.); pablop@iastate.edu (P.P.); 2 Merck Animal Health, 1102 Southern Hills Dr #101, Ames, IA 50010, USA; mark.mogler@merck.com (M.M.); estrait@dalananimalhealth.com (E.S.)

**Keywords:** challenge, live exposure, parainfluenza, porcine respirovirus 1, nursery, respiratory, replicon particle, swine, vaccine

## Abstract

Parainfluenza viruses are a common cause of respiratory illness in many species. In this study, experimental, alphavirus-derived RNA particle vaccines either with or without adjuvant were evaluated against porcine parainfluenza virus 1 (PPIV1) challenge and compared to live virus exposure. Groups of ten, three-week-old pigs were vaccinated intramuscularly with an adjuvanted RNA particle (RPAdj/C) or non-adjuvanted RP (RP/C) or administered an intranasal live exposure (LE/C) dose of PPIV1 at 0- and 21-days post vaccination (DPV) followed by challenge with PPIV1 at 40 DPV. In addition, two groups were included as non-vaccinated, non-challenged (NV/NC) and non-vaccinated, challenged (NV/C) controls. Intranasal virus exposure and RP vaccination, regardless of adjuvant, reduced PPIV1 shedding in nasal swabs by 5 days post inoculation (DPI). All vaccinated or exposed pigs seroconverted as shown by enzyme-linked immunosorbent assay and serum virus neutralization. The antibody isotype detected in bronchoalveolar lavage fluid (BALF) LE/C was predominantly IgA while RP vaccination induced an IgG response. Reduced PPIV1 antigen was observed in the LE/C, RP/C and RPAdj/C groups in lung, trachea, or nasal turbinate epithelium. Additionally, the RPAdj vaccine significantly reduced nasal shedding compared to NV/C pigs although not as much as LE/C pigs. These results suggest vaccination could mitigate PPIV1 infection in commercial systems.

## 1. Introduction

Porcine parainfluenza virus 1 (PPIV1) is a recently characterized swine respiratory agent in the family *Paramyxoviridae* and genus *Respirovirus*. Other related viruses in the same genus include human parainfluenza virus 1 (HPIV1), human parainfluenza virus 3 (HPIV3), bovine parainfluenza virus 3 (BPIV3), and Sendai virus (SeV) [1]. PPIV1 was originally detected in samples collected from slaughtered swine in 2013 in China [2]. PPIV1 is considered widespread in swine and has been reported in North America [3,4], South America [5], and Europe [6].

Field observations have suggested PPIV1 can cause respiratory disease in the absence of other swine respiratory pathogens [7]. However, these observations have not been repeated under controlled experimental conditions [8] or during swine influenza (IAV-S) coinfection studies [9]. Further studies are necessary to establish whether clinical respiratory disease is correlated with differences of PPIV1 strains, since there is preliminary evidence suggesting genetic diversity among PPIV1 detected in the United States (U.S.) [4]. It is also unclear if and how bacterial coinfection with PPIV1 affects the severity of clinical disease. Despite its current ambiguous role in the porcine respiratory disease complex (PRDC), there is an interest in understanding the potential impact of vaccines against PPIV1.

Parainfluenza viruses are currently included in combination vaccines including distemper, adenovirus, parainfluenza, and parvovirus (DHPP) administered to dogs and bovine respiratory syncytial virus, infectious bovine rhinotracheitis virus, and BPIV3 vaccines administered to calves. Developing effective parainfluenza virus (PIV) vaccines has historically been a challenge as the primary immune response is rarely sterilizing [10,11]. The development of HPIV vaccines was hindered in the late 1960s due to the formalin-inactivated vaccines not providing protection despite a systemic, neutralizing response [12,13]. Several HPIV vaccines are in development, but none are commercially available [11]. Often multiple reinfections are necessary to confer lasting immunity, especially between different serotypes [14].

To date, studies that have investigated the effectiveness of vaccines or the immunogenicity of PPIV1 after natural exposure have not been conducted in swine. Alphavirus RNA particle (RP) vaccines have been developed for a variety of swine viruses including influenza [15] and porcine epidemic diarrhea virus (PEDV) [16]. Replicon particle vaccines are generated by co-transfecting the alphavirus replicon RNA (replicase), a transgene encoding the antigen RNA of interest, and helper RNAs containing the alphavirus structural genes. RNA particle vaccines are an alternative to whole-inactivated vaccines as they facilitate the intracellular expression of antigen mediated by the alphavirus replicon RNA, allowing for the potential to stimulate both humoral and cellular immune responses through CD4+ and CD8+ T lymphocytes [17]. In this study, an alphavirus RP vaccine was tested against homologous PPIV1 challenge either with or without adjuvant and compared to natural exposure.

## 2. Materials and Methods

All laboratory and animal procedures were first reviewed by the Iowa State University Institutional Biosafety Committee (ref #18-041) and approved by Institutional Animal Care and Use Committee prior to initiating this study (ref #18-161).

### 2.1. PPIV1 Cell Culture and Inoculation

A PPIV1 isolate USA/IA/84915LG/2017 (IA17; GenBank MG75374) was grown in swine testicular (ST) cells (ATCC CRL-1746™) following the previously described protocol with a slight modification [18]. Briefly, ST cells were maintained in Earle’s minimum essential medium (MEM; Thermo Fisher Scientific, Waltham, MA, USA) supplemented with 10% fetal bovine serum (FBS; Atlas Biologicals, Fort Collins, CO, USA) with 1× antibiotics (ABS) consisting of 100U penicillin, 100 µg/mL streptomycin, 2 mM L-glutamine, 50 µg/mL gentamycin, (Gibco™, Waltham, MA, USA), and 0.25 µg/mL amphotericin B (Sigma-Aldrich, St. Louis, MO, USA). When ST cells reached 95% confluency (~2 days), the cells were inoculated with PPIV1 at a multiplicity of infection of 0.01 in post inoculation medium (PIM) consisting of minimum essential medium (MEM), 1 µg/mL tosyl phenylalanyl chloromethyl ketone (TPCK)-treated trypsin, and 1× ABS. The propagated virus was titrated by inoculating serially diluted virus into ST cells grown in 96-well plates (5 wells/dilution). The dilution plates were fixed and visualized using a horseradish peroxidase (HRP) stain as described previously [4]. The virus titer expressed as 50% tissue culture infectious dose per milliliter (TCID_50_/mL) was calculated using the Reed–Muench method [19].

### 2.2. Animals

Fifty, three-week-old pigs were sourced from a commercial breed-to-wean herd from dams confirmed PPIV1 antibody negative using a whole virus enzyme-linked immunosorbent assay (wvELISA) as previously described [20]. Pigs also tested negative for PPIV1 RNA by real-time reverse-transcriptase polymerase chain reaction (RT-rtPCR) at the Iowa State University Veterinary Diagnostic Laboratory (ISU VDL) prior to leaving the source farm [4]. After arrival at the ISU research facility, nasal swabs were screened negative by RT-rtPCR for influenza A virus (IAV) and PPIV1 RNA and by rtPCR for *Mycoplasma hyopneumoniae* (MHP) DNA. Additionally, serum was screened by RT-rtPCR for porcine reproductive and respiratory syndrome virus (PRRSV) and rtPCR for porcine circovirus-2 (PCV2) viremia and tested negative. All PCR assays were conducted according to ISU VDL standard operating procedures. All pigs were administered an intramuscular (IM) injection of ceftiofur crystalline-free acid (Excede^®^; Zoetis, Florham Park, NJ, USA) per label instructions upon arrival.

### 2.3. Experimental Design

Pigs (*n* = 50) were randomized by weight, assigned to treatments, and allocated into five rooms with 10 pigs per room. The experimental RP vaccines were formulated as a mixture of two RNA particles expressing either the hemagglutinin-neuraminidase or fusion surface glycoproteins. One treatment group received the RP alone while the other received a vaccine with an oil-in-water adjuvant. Treatments consisted of a non-vaccinated, non-challenged group (NV/NC); a non-vaccinated, challenged group (NV/C); an RP vaccinated, challenged group (RP/C); an adjuvanted RP vaccinated, challenged group (RPAdj/C); and a live exposure, challenged group (LE/C). The pigs in the RPAdj/C and RP/C groups received a 2 mL IM dose of their respective RP vaccine in the neck. The adjuvant was added in a 1:1 *v:v* ratio to the RPAdj vaccine immediately prior to administration. The RP and RPAdj vaccines were administered at 0- and 21-days post vaccination (DPV). Pigs in the LE/C group were intranasally (IN) exposed with 2 mL of IA17 at a concentration of 6.3 × 10^4^ TCID_50_/mL divided equally between each nostril at 0 and 21 DPV (Table 1). NV/C, RP/C, RPAdj/C, and LE/C groups were challenged at 40 DPV with PPIV1 virus isolate IA17 at the same concentration (6.3 × 10^4^ TCID_50_/mL) under telazol, ketamine, and xylazine sedation as previously described [18]; 2 mL was administered intratracheally, and 2 mL was divided evenly between each nostril. Both nares were swabbed with a polyester-tipped sterile applicator (Fisher Scientific, Waltham, MA, USA) and placed into 2 mL of plain MEM without any additives. All pigs were humanely euthanized and necropsied at 45 DPV (Table 1) by intravenous pentobarbital overdose (Fatal-Plus^®^; Vortech Pharmaceuticals, Dearborn, MI, USA). The following samples were collected from all groups: serum at −3, 8, 15, 21, 28, 35, 40, and 45 DPV; oral fluid was collected on 0, 15, 20, 25, 28, 32, 41, 43, and 45 DPV; and nasal swabs at −7, 0, 21, 40, 41, 42, 43, 44, and 45 DPV. At necropsy, formalin-fixed tissues were collected, including four sections of lung (right cranial, right caudal, accessory, diaphragmatic), trachea, and nasal turbinate (NT). Several fresh samples were collected for quantitative RT-PCR (RT-qPCR) including left cranial lung lobe, tracheal swab (TS), NT, and bronchioalveolar lavage fluid (BALF).

### 2.4. Clinical Observations

Body weights were recorded for each pig on arrival (−3 DPV), prior to vaccination (21 DPV), prior to challenge (40 DPV), and prior to necropsy (45 DPV) (Table 1). Average daily weight gain (ADWG) was computed by subtracting the initial weight from the final weight and dividing by the intervening number of days on trial. Rectal temperatures and clinical observation scores were collected daily from 40 to 45 DPV. Clinical observation scores consisted of coughing (0 = absent, 1 = present) and respiratory distress scores (0 = normal; 1 = mild, slow to move, head down; 2 = moderate dyspnea, notable increased respiratory rate; 3 = severe dyspnea, notable increase respiratory rate, abdominal breathing).

### 2.5. Sample Processing

Fresh samples were processed per ISU VDL standard operating procedures. Briefly, a ten percent weight by volume (*w/v*) homogenate solution was prepared by placing 1 g of tissue into 10 mL of Earle’s balanced salt solution (Earle’s; Sigma Aldrich, St. Luis, MO, USA). For NT, a disposable tissue grinder system (Thermo Fisher Scientific, Waltham, MA, USA) using 0.5 ± 0.1 g of tissue placed into 5 mL of Earle’s and ground for 30 s to create a 10% *w/v* homogenate. Nasal swabs (NS) and TS were placed into 2 mL of plain MEM without additives. BALF samples were collected from lungs at necropsy as previously described [21] and required no additional sample processing before RNA extraction [22].

### 2.6. Nucleic Acid Extraction

Total nucleic acid was extracted with the 5× Ambion^®^ MagMax™ 96 Viral RNA Kit (Thermo Fisher Scientific, Waltham, MA, USA) and Kingfisher 96^®^ magnetic particle processor (Thermo Fisher Scientific, Waltham, MA, USA) using a protocol slightly modified from the manufacturer’s instructions. Specifically, 100 µL of the sample, 240 µL of lysis binding buffer, 300 µL of wash I, and 450 µL of wash II were used and nucleic acid eluted into 90 µL of elution buffer. An in-house exogenous internal positive control (XIPC) was added to the lysis buffer to monitor for internal inhibition as described previously with minor modifications [23].

### 2.7. PPIV1 RT-qPCR Assay

An RT-qPCR targeting the nucleoprotein was used to detect PPIV1 viral RNA as previously described [4]. Signal amplification was produced using a 7500 Fast thermocycler (Applied Biosystems, Foster City, CA, USA) to 40 cycles. Fluorescence was produced by a probe containing a 5′ FAM fluorophore and 3′ Iowa Black^®^ quencher. All cycle thresholds were converted to genomic copies per mL (GC/mL) based on a standard curve developed at the ISU VDL through serial dilutions of a known quantity of PPIV1 RNA template produced by in vitro transcription.

### 2.8. Detection of Viral Antigen by Immunohistochemistry (IHC)

PPIV1 antigen was visualized on sections of formalin-fixed and paraffin-embedded (FFPE) lung as previously described [18]. Briefly, 4 µm thick tissue sections were heated at 72 °C in 150 µL of bond dewaxing solution for 30 min. The tissues were rehydrated with alcohol and blocked with hydrogen peroxide for 7 min. The slides were then stained with PPIV1 polyclonal antibody for 45 min at 1:100 dilution and then anti-mouse horse radish peroxidase (HRP) conjugated secondary antibody for 25 min. Next, the slides were incubated with 3′3-diaminobenzene (DAB) chromagen for 10 min. The slides were counterstained with hematoxylin for 5 min, cover-slipped, and analyzed by a veterinary pathologist blinded to treatment groups.

### 2.9. Gross and Microscopic Lesions

A veterinary pathologist blinded to treatment groups scored gross lung lesions, microscopic lesions, and IHC staining. The total amount of lung affected by consolidation was estimated by adding the weighted proportions of each lung lobe relative to the total lung surface area [24]. Microscopic lung and trachea lesions were scored as previously described [18]. Microscopic lung lesion scores were computed into a composite lower respiratory tract (LRT) score. The LRT score ranged from 0 to 22 and was composed of the following individual scores: interstitial pneumonia (0–4), peribronchiolar cuffing (0–4), airway epithelial necrosis (0–4), suppurative bronchiolitis (0–4), epithelial microabscesses (0–3), and alveolar edema (0–3). The composite trachea score ranged from 0 to 8 and consisted of tracheitis (0–4) and trachea epithelial necrosis (0–4). Epithelial IHC staining for PPIV antigen was evaluated for the lung (0–4), trachea (0–4), and turbinate (0–4).

### 2.10. Serum Virus Neutralization Assay

Serum virus neutralization (SVN) antibody titers were evaluated as previously described [18]. Briefly, 96-well plates (Thermo Fisher Scientific, Waltham, MA, USA) were seeded with MK2 cells (ATCC CCL-7™) and incubated at 37 °C in 5% CO_2_ until they reached 95% confluency (1–2 days) in M199 medium (Thermo Fisher Scientific, Waltham, MA, USA) containing 1% equine serum (Sigma Aldrich, St. Louis, MO, USA), 100 U/mL penicillin, and 100 µg/mL streptomycin (Thermo Fisher Scientific, Waltham, MA, USA). Sera were heat inactivated for 30 min at 56 °C and serially diluted in two-fold increments starting at 1:10 in PIM. An equal volume of PPIV1 challenge virus (IA17) was added to each of the diluted sera at a concentration of 200 TCID_50_/well, equivalent to 4 × 10^3^ TCID_50_/mL. Infection and PIM-only control columns were included on each plate. The cells were washed 2× with pre-warmed MEM before 100 µL of the serum–virus mixture was added to the cells and incubated for 2 h. Next, the serum–virus mixture was removed and the cells were again washed 2× with 125 µL of MEM before PIM was added to the cells and incubated for 72 h at 37 °C in 5% CO_2_. The cell plates were fixed with pre-chilled 80% acetone at −20 °C for 15 min and dried at room temperature (RT) for 10 min. The plates were either stored at −20 °C for later use or stained immediately by immunocytochemistry (ICC) as previously described [18]

### 2.11. Detection of PPIV Antibodies by wvELISA

The presence of antibodies in serum and BALF were evaluated with PPIV1 wvELISA as described previously [20]. Briefly, 500 mL of virus was grown to a titer of 10^6.5^ TCID_50_/mL as described above in ST cells. The supernatant was clarified at 4200× *g* to remove cellular debris and ultracentrifuged at 140,992× *g* for 3 h. The pellet was washed twice and resuspended in 100 µL 1× phosphate-buffered saline at a pH of 7.4 (PBS; Thermo Fisher Scientific, Waltham, MA, USA). The plates were coated with 100 µL per well at a dilution of 1:200 in polystyrene 96 well ELISA plates at 4 °C for 16 h (Nunc, Maxisorp; Thermo Fisher Scientific, Agawam, MA, USA). The optimum dilution (1:200) was determined using a checkerboard titration based on known status antibody positive and negative samples to maximize signal while minimizing background noise. The plates were then washed 5× with 300 µL of PBST (0.1% Tween 20 in PBS), blocked with 300 µL of a blocking solution containing 1% bovine serum albumen (BSA, West Grove, PA, USA) and incubated at RT for 2 h. The plates were dried and stored at 4 °C for later use.

Serum samples were tested at 1:100 dilution in sample diluent containing 40% newborn calf serum (Thermo Fisher Scientific, Waltham, MA, USA), incubated at RT for 1 h, washed 5× with PBST, and 100 µL of HRP-conjugated goat anti-pig IgG (Fc) antibody (Bethyl Laboratories, Inc., Montgomery, TX, USA) diluted 1:20,000 in 1% BSA solution were added to each well. BALF samples were tested for IgG and IgA by wvELISA but adapted for the sample matrix. The BALF samples were diluted 1:50 in blocking buffer containing 40% newborn calf serum. The reaction was visualized by adding 100 µL of tetramethylbenzidine-hydrogen peroxide (TMB) substrate solution for 10 min at RT. Stop solution (100 μL) was added to each well (Surmodics, Inc., Eden Prairie, MN, USA). The sample to positive ratio was calculated using the following equation:

$$SP\;Ratio:\frac{\left(sample\;OD-negative\;control\;mean\;OD\right)}{\left(positive\;control\;mean\;OD-negative\;control\;mean\;OD\right)}$$

### 2.12. Statistical Analysis

Data were analyzed using commercially available software (SAS^®^ Version 9.4; SAS^®^ Institute, Inc., Cary, NC, USA). Normality and other model assumptions were assessed using the PROC UNIVARIATE procedure and diagnostic residual plots available in the PROC-MIXED package. Rectal temperatures, SVN antibody titers in serum, wvELISA S/P ratios, and PPIV1 viral loads in nasal swabs were analyzed with the PROC-MIXED procedure using the most appropriate covariance structure for the data based on comparison of corrected Akaike’s information criterion values for model fit. Serum wvELISA IgG S/P values were transformed by the natural logarithm prior to analysis. Kruskal–Wallis and post hoc Dwass–Steel–Critchlow–Fligner multiple comparison tests available in PROC NPAR1WAY were used to analyze S/P values from the wvELISA BALF samples, necropsy RT-qPCR samples, histology scores, and IHC scores.

## 3. Results

### 3.1. Average Daily Gain

Significant differences in average daily weight gain (ADWG) between treatment groups were not observed during −3 to 21 DPV, −3 to 40 DPV, or at −3 to 45 DPV necropsy.

### 3.2. PPIV1 Detection of Viral Shedding in NS by RT-qPCR

**Figure 1:**
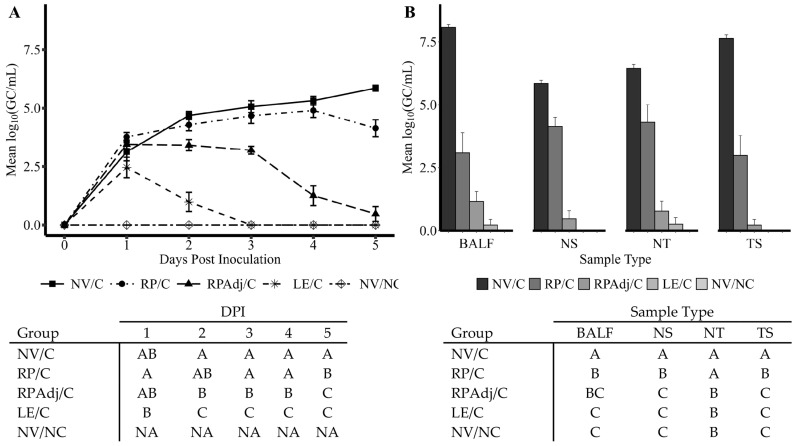
(**A**) Porcine parainfluenza virus-1 (PPIV1) shedding in NS detected by RT-qPCR. The data are expressed as mean log_10_ genomic copies/mL (GC/mL). The level and duration of shedding was most reduced in the LE/C group with the RPAdj/C group also demonstrating significant reduction. The effect in the RP/C group was more delayed and did not appear until 5 DPI. (**B**) PPIV1 viral load detected by RT-qPCR in tissue samples collected at 5 DPI necropsy. The data are expressed as mean log_10_ (GC/mL) of the tissue homogenate. Significant reductions in PPIV1 RNA were observed in the vaccinated and exposed groups relative to the NV/C group after challenge with IA17. The RP/C group demonstrated reduced mean viral loads in all samples, but the LE/C and RPAdj/C groups had reductions comparable to the NV/NC group (*p* < 0.05). Connecting letters–tables shown under each figure are provided to indicate significance at *p* < 0.05. Nasal swabs (**A**) were analyzed using a linear mixed model excluding the NV/NC group, and necropsy samples were analyzed by a Kruskal–Wallis test and a post hoc Dwass–Steel–Critchlow–Fligner test if significant. Groups with different letters are significantly different at *p* < 0.05. PPIV1: porcine parainfluenza virus 1; NV/NC: non-vaccinated/non-challenged; NV/C: non-vaccinated/challenged; RPAdj/C: RNA particle adjuvanted vaccine/challenged; RP/C: RNA particle vaccine/challenged; LE/C: live exposed/challenged; BALF: bronchoalveolar lavage fluid; NS: nasal swab; NT: nasal turbinate, TS: tracheal swab; NA: not analyzed to facilitate fit of the statistical model.

Based on detection of PPIV1 nucleic acid in nasal swabs by RT-qPCR, the PPIV1 LE and RPAdj vaccination reduced the duration and quantity of viral shedding post challenge with IA17 (Figure 1A). The duration of shedding was the shortest in the LE/C group followed by the RPAdj/C group, respectively. Shedding was undetectable from all pigs in the LE/C group on 3–5 DPI. In contrast, shedding in the RP/C group was not significantly different from the NV/C group on 2–4 DPI (*p* > 0.05) but PPIV1 GC/mL were significantly lower in the RP/C group on 5 DPI (*p* < 0.05). The RPAdj/C group had significantly decreased PPIV1 GC/mL in NS on 3–5 DPI compared to the RP/C group. However, PPIV1 GC/mL were not significantly different in the LE/C and RPAdj/C groups at 5 DPI. The highest levels of PPIV1 RNA in NS were detected in the NV/C group at 2–5 DPI, averaging between 10^4.7^ and 10^5.9^ GC/mL on 2 and 5 DPI (Figure 1A). The replication kinetics of PPIV1 in NS are consistent with previous challenge experiments [18].

### 3.3. PPIV1 Detection in Tissues by RT-qPCR

A significant reduction in GC/mL was observed in the RP/C, RPAdj/C, and LE/C groups at 5 DPI compared with NV/C group (Figure 1B). No significant difference was observed between the LE/C, RPAdj/C, and NV/NC groups in BALF, NS, NT, and TS samples (*p* > 0.094). Significantly reduced viral loads were detected in NS, NT, and TS in the RPAdj/C group compared to the RP/C group; however, this difference was not observed in BALF (*p* = 0.399). The RP/C vaccinated group demonstrated significantly lower PPIV1 RNA in BALF, NS, and TS compared to the NV/C. Interestingly, significant differences were not detected between RP/C and NV/C in NT samples (*p* = 0.11), although virus levels were lower in the RP/C group.

### 3.4. Macroscopic Lung Lesions

Macroscopic lung lesions were mild to absent in all groups, consistent with previous PPIV1 infection studies [18]. Less than 5% of lung surface area in all groups were affected or demonstrated consolidation.

### 3.5. Microscopic Lesions and Immunohistochemistry

**Figure 2:**
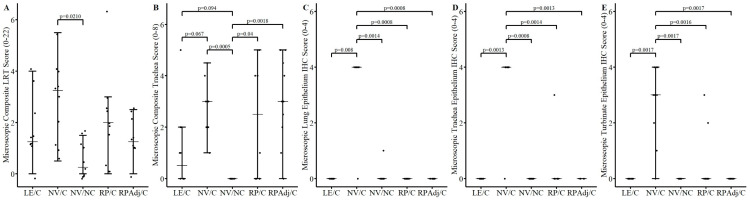
Median composite PPIV1 microscopic LRT, microscopic trachea lesion, and IHC scores by treatment group at 5 DPI necropsy. Error bars represent 25th, 50th, and 75th quartiles, respectively. Points are jittered horizontally to prevent overplotting. All data were analyzed with a Kruskal–Wallis test and a post hoc Dwass–Steel–Critchlow–Fligner test if significant. Significance brackets are provided if *p* < 0.1. (**A**) Median composite microscopic LRT lesion scores by treatment group. Composite LRT scores were slightly increased in the NV/C group; however, no significant differences were observed in the other challenge groups. (**B**) Median microscopic trachea lesion scores. Composite scores were significantly increased in the NV/C, RP/C and RPAdj/C groups compared to the NV/NC group. No significant difference was observed between the LE/C and NV/NC groups. Median microscopic PPIV1 IHC scores in the (**C**) lung, (**D**) trachea, (**E**) NT epithelium. Reduced PPIV1 signal was consistently observed in the LE/C, NV/NC, RP/C, and RPAdj/C groups across tissue sections. PPIV1: porcine parainfluenza virus 1; LRT: lower respiratory tract; IHC: immunohistochemistry; DPI: days post inoculation; NV/NC: non-vaccinated/non-challenged; NV/C: non-vaccinated/challenged; RPAdj/C: RNA particle adjuvanted vaccine/challenged; RP/C: RNA particle vaccine/challenged; LE/C: live exposed/challenged.

**Figure 3:**
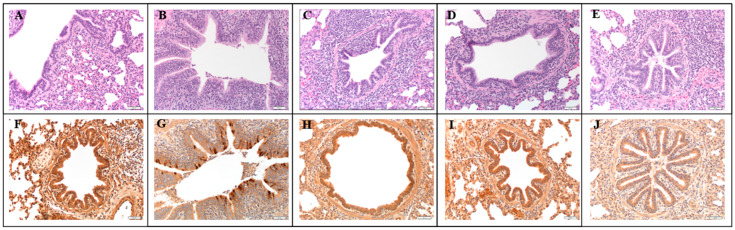
Representative microscopic histopathology lesions (**A**–**E**) and IHC staining (**F**–**J**) from fixed lung at 200× magnification. Each column corresponds to a treatment group. (**A**) NV/NC, no microscopic lesions (**B**–**E**) corresponding to NV/C, RP/C, RPAdj/C, and LE/C groups, respectively. Mild epithelial proliferation and peribronchiolar cuffing was observed in all groups, but most pronounced in the NV/C group. (**F**) NV/NC, no PPIV1 signal detected, (**G**) NV/C, abundant PPIV1 specific IHC signal in the bronchiolar epithelium, (**H**–**J**) corresponding to RP/C, RPAdj/C, and LE/C groups, respectively, with no or minimal PPIV1 signal detected. PPIV1: porcine parainfluenza virus 1; IHC: immunohistochemistry; NV/NC: non-vaccinated/non-challenged; NV/C: non-vaccinated/challenged; RPAdj/C: RNA particle adjuvanted vaccine/challenged; RP/C: RNA particle vaccine/challenged; LE/C: live exposed/challenged.

The composite LRT score was significantly higher in the NV/C group compared to the NV/NC group (Figure 2A, *p* = 0.021). However, significant differences in microscopic lung scores between the other groups were not observed (*p* > 0.15). Microscopic lung lesions were characteristic of an epitheliotropic virus and consistent with prior PPIV1 inoculation studies but were mild overall [18]. Microscopic lung lesions were minimal or not observed in the NV/NC group (Figure 3A). Peribronchiolar cuffing comprised of a mononuclear infiltrate of lymphocytes and monocytes with mild epithelial proliferation was observed in the NV/C group (Figure 3B) and to a lesser degree in the RP/C (Figure 3C), RPAdj/C (Figure 3D), and LE/C (Figure 3E) groups. The tracheal lesions score in the RP/C and RPAdj/C groups were significantly higher relative to the NV/NC group (Figure 2B; *p* < 0.04). In contrast, microscopic trachea lesion scores observed in pigs from the LE/C group were not statistically different from the NV/NC (*p* = 0.094) suggesting prior PPIV1 exposure may have reduced trachea lesions.

Abundant IHC staining was observed in the NV/C group in the lung epithelium (Figure 2C and Figure 3G) and also in the trachea (Figure 2D) and NT (Figure 2E). Minimal viral antigens were detected in the lung (Figure 2C; *p* < 0.008), trachea (Figure 2D; *p* < 0.0014), and NT (Figure 2E; *p* < 0.0017) epithelium by PPIV IHC in the NV/NC and vaccinated groups relative to the NV/C group. Representative lung IHC micrographs taken at 200× magnification further illustrate the decrease in IHC staining shown in the RP/C (Figure 3H), RPAdj/C (Figure 3I), and LE/C (Figure 3J) groups.

### 3.6. Detection of IgG Antibody by wvELISA in Serum

**Figure 4:**
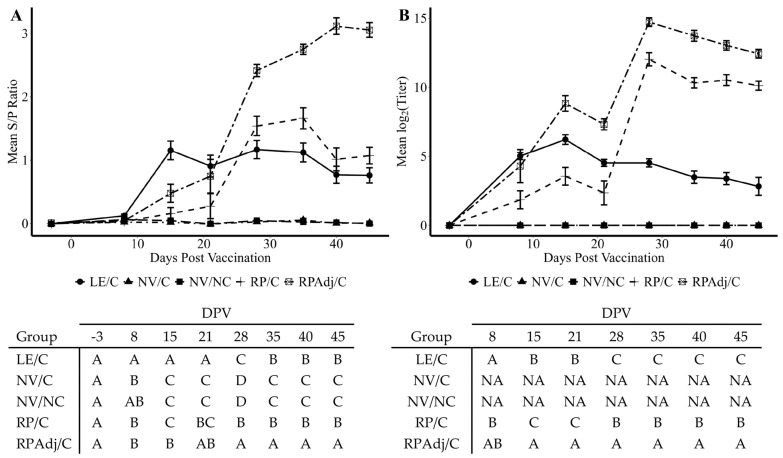
PPIV1 antibody detection demonstrated as (**A**) mean wvELISA sample to positive (S/P) ratios of circulating anti-PPIV1 IgG antibodies in serum; (**B**) mean log_2_ transformed SVN titers of circulating anti-PPIV1 neutralizing antibodies in serum. SVN titers are expressed as the reciprocal of the highest dilution with ≥95% reduction in infectivity. Significantly higher antibody levels were observed in the RPAdj/C and RP/C groups compared to the LE/C group by wv-ELISA and SVN at different days post vaccination. All data were analyzed using a linear mixed model with the most appropriate covariance structure determined by Akaike’s information criterion. Connecting letters reports shown under each figure are provided to indicate significance at *p* < 0.05. Groups with different letters are significantly different. PPIV1: Porcine parainfluenza virus 1; wvELISA: whole virus enzyme linked immunosorbent assay; SVN: serum virus neutralization; NV/NC: non-vaccinated/non-challenged; NV/C: non-vaccinated/challenged; RPAdj/C: RNA particle adjuvanted vaccine/challenged; RP/C: RNA particle vaccine/challenged; LE/C: live exposed/challenged; NA: Not analyzed to facilitate fit of the statistical model.

Systemic antibodies were significantly higher in the RPAdj/C groups after the second vaccination at 21 DPV compared to all other groups (*p* < 0.0001) suggesting an anamnestic response to the vaccine demonstrated at 28, 35, 40, and 45 DPV (Figure 4A). Detectable IgG antibody levels were measured in the LE/C group by 8 DPV compared to the RPAdj/C, RP/C, and NV/C groups (*p* < 0.02); however, the LE/C group lacked a significant difference compared to the NV/NC group (*p* = 0.14). Increased PPIV1 serum antibodies were also significantly higher in the RP/C group compared to the LE/C group at 28 DPV (*p* = 0.04). However, significant differences were not observed between the LE/C and RP/C groups on 35, 40, and 45 DPV (*p* = 0.06). Comparable mean S/P ratios were detected between the NV/NC and NV/C groups for the study duration (*p* > 0.1).

### 3.7. Serum Virus Neutralization Antibody Titers

A similar trend was observed in mean SVN antibody titers compared to the wvELISA serology S/P results (Figure 4B). Neutralizing antibody was detected at 8 DPV in the LE/C, RP/C, and RPAdj/C groups. A significantly higher titer was observed in the RPAdj/C group relative to the LE/C group by 15 DPV (*p* = 0.002) in contrast to S/P ratios being higher in the LE/C group relative to the RPAdj/C group by wvELISA. The SVN titers were significantly lower in the RP/C group compared to the RPAdj/C group at 15–45 DPV (*p* < 0.02) and trended lower at 8 DPV (*p* = 0.09) although not significant. Both RP/C and RPAdj/C titers markedly increased one week after the second vaccine dose at 28 DPV suggesting an anamnestic response; a corresponding increase was not observed in the LE/C group after the second PPIV1 intranasal exposure. Differences in mean SVN titers were more pronounced between the RP/C and LE/C group after administration of the second vaccine dose on 28–45 DPV than was detected by wvELISA. No animals from the NV/NC and NV/C groups had detectible SVN titers for the study duration, and both groups were excluded from the analysis to improve the statistical model.

### 3.8. Detection of IgG and IgA in BALF

**Figure 5:**
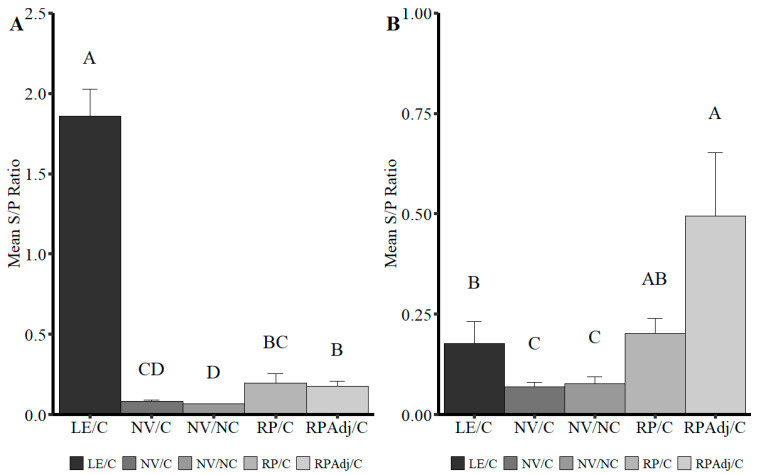
Anti-PPIV1 antibody levels specific for (**A**) IgA or (**B**) IgG detected in BALF by PPIV1 wvELISA. Significantly higher mean IgA antibody levels were detected in the LE/C BALF compared to the other groups. Trace amounts of IgA were observed in the RPAdj/C and RP/C vaccinated groups. In contrast, increased levels of IgG were detected in the RP/Adj group with slightly lower amounts detected in the RP/C and LE/C groups. All data were analyzed with a Kruskal-Wallis test and a post hoc Dwass–Steel–Critchlow–Fligner test if significant. Different letters indicate significant differences at *p* < 0.05. PPIV1: porcine parainfluenza virus 1; NV/NC: non-vaccinated/non-challenged; NV/C: non-vaccinated/challenged; RPAdj/C: RNA particle adjuvanted vaccine/challenged; RP/C: RNA particle vaccine/challenged; LE/C: live exposed/challenged.

Higher levels of BALF IgA and IgG were detected in the LE/C and RPAdj/C groups, respectively, which may correspond to either the route of vaccination, route of exposure, or type of vaccine platform (Figure 5A,B). Significantly lower levels of IgA were detected in the BALF of the RP/C and RPAdj/C compared to the LE/C group (Figure 5A; *p* = 0.0015), but still significantly higher than the NV/NC group (*p* < 0.003). All three vaccinated or exposed groups (RPAdj/C, RP/C, and LE/C) had significantly higher IgG S/P ratios in BALF relative to the NV/NC and NV/C groups (*p* < 0.017). Levels of IgG in BALF followed the opposite trend compared to IgA levels with the highest S/P ratio detected in the RPAdj/C (Figure 5B; *p* < 0.013); however, no significant difference was observed in median IgG S/P ratios between the LE/C and RP/C groups (*p* = 0.91).

## 4. Discussion

This study investigated the efficacy of experimental RNA particle vaccines against PPIV1 in nursery-age piglets in comparison to live PPIV1 intranasal exposure. Field reports suggest that PPIV1 can cause respiratory disease in the absence of other swine respiratory pathogens [7]. Although these findings have not been replicated experimentally, a PPIV1 vaccine could benefit herds with a history of PPIV1-associated respiratory disease characterized by persistent coughing. It is still unknown whether natural exposure against PPIV1 would protect against reinfection, even though prior PPIV1 experimental inoculation studies demonstrated serum neutralizing titers as early as one week post challenge and increased serum IgG between 10 and 14 DPI [18].

The development of vaccines against PIVs and other paramyxoviruses can be challenging. Natural IgA immunity to PIVs is considered short-lived, with multiple infections needed to confer adequate protection [13]. Vaccination of children in the 1960s with formalin-inactivated PIV and respiratory syncytial virus (RSV) resulted in incomplete immunity and enhanced disease [25]. It was discovered with measles virus (a related paramyxovirus) that formalin inactivation led to an overwhelming CD4+ type 2 response and the induction of non-protective but biologically active antibodies [26]. Preclinical data indicate that live attenuated vaccines do not predispose to severe LRT illness as observed with formalin-inactivated vaccines [27]. However, live, attenuated vaccines present a risk of reversion to virulence or may recombine with circulating field strains. Alphavirus-derived RP vaccines stimulate both the humoral and cellular immune responses in both CD4+ and CD8+ T lymphocytes [17], making them a viable alternative to live, attenuated vaccines. Additionally, virulent virus particles cannot be produced from replication-deficient RP vaccines.

In the current study, RP vaccination, with and without adjuvant, showed a significant reduction in viral loads in NS, BALF, and TS compared to the NV/C group at 5 DPI (Figure 1A,B). The LE/C group was included as a gold standard measure for comparison of vaccine efficacy. The duration of PPIV1 shedding in NS was comparable between the LE/C and RPAdj/C group with only a 2-day difference between the two groups. The adjuvant appeared to have a significant impact on vaccine efficacy as the duration of shedding and viral loads in tissues were lower relative to the RP/C group that lacked adjuvant. However, by 5 DPI, virus load detected in the RP/C pigs was significantly lower compared to the NV/C group in NS, BALF, and TS suggesting some efficacy. Similar results were found with other PIV RP vaccines tested in hamsters [28] and mice [29]. These studies showed that the RP vaccination reduced overall viral loads in the lung after IM and IN vaccination in hamsters and mice. The results were also comparable to IAV-S RP vaccination in pigs with reduced shedding found in NS on 5 DPI after challenge or 45 DPV [30]. Interestingly, the role of adjuvant in promoting an immune response was not investigated in any of these prior studies.

The onset of IgG detection by wvELISA was approximately 15 days and consistent with prior inoculation experiments (Figure 4A) [18]. SVN titers were observed in the LE/C, RPAdj/C, and RP/C groups as early as 8 DPV (Figure 4B). IgG levels were highest in the RPAdj/C group, followed by the LE/C and RP/C groups after the boost dose of vaccine. The onset of IgG production in the LE/C group was consistent with previous PPIV1 challenge studies which consistently showed seroconversion to ELISA antibody at approximately 10–14 DPI [18]. Mucosal IgA was the prevailing antibody isotype detected in BALF collected at necropsy from the LE/C group (Figure 5A) while IgG was detected at a higher concentration in the BALF collected from the RPAdj/C group (Figure 5B). Antibody isotype may play a significant role protecting against PPIV1 infectivity. Previous work in IAV demonstrated increased ability of IgA to inhibit cellular release compared to IgG. Additionally, both trimeric and tetrameric IgA demonstrated stronger anti-IAV activity than monomeric IgA and IgG [31,32,33,34]. Interestingly, the intramuscular-delivered RP and RPAdj vaccines demonstrated high levels of IgA compared to the non-vaccinated pigs suggesting some potential IgA protection against infection with these vaccines (Figure 5A). However, inactivated vaccines may require booster vaccination to maintain protection [35].

Two IN exposures of PPIV1 resulted in robust immunity with minimal clinical signs and microscopic lesions. PIVs have been used as vaccine vectors for more virulent pathogens such as respiratory syncytial virus in humans [36] and highly pathogenic avian influenza in poultry [37]. There is previously published research investigating canine PIV5 as a potential bivalent vaccine vector for IAV-S [38]. However, using a host-adapted PIV strain may result in improved immunity due to increased replication, resulting in better protection. PPIV could therefore be used as a vector for vaccines against other swine respiratory pathogens.

Based on this specific experimental design, the results of this vaccine-challenge study suggest that vaccination with an adjuvanted PPIV1 RP vaccine reduced virus shedding, replication in tissues, and IHC signal comparable to live exposure at 5 DPI. Additionally, the RP vaccine significantly reduced the duration of shedding in NS, although not as brief as demonstrated by pigs administered live intranasal PPIV1 exposure. If the RP vaccine performance is mimicked in the field, vaccination with a similar RNA particle vaccine formulation may prove beneficial for herds with a history of PPIV1 clinical disease by reducing viral loads. However, more studies are necessary to confirm the protective ability of PPIV1 RP vaccines.

## Figures and Tables

**Table 1:** Experimental design, group assignments, and vaccination schedule.

				Vaccination		Challenge	
Group	*N*	Vaccine Group	Vaccination Route	Priming Dose	Boost Dose	Challenge	Necropsy
1	10	NV/NC	IM	0 DPV	21 DPV	40 DPV/0 DPI	45 DPV/5 DPI
2	10	NV/C	IM	0 DPV	21 DPV	40 DPV/0 DPI	45 DPV/5 DPI
3	10	RPadj/C	IM	0 DPV	21 DPV	40 DPV/0 DPI	45 DPV/5 DPI
4	10	RP/C	IM	0 DPV	21 DPV	40 DPV/0 DPI	45 DPV/5 DPI
5	10	LE/C	IN	0 DPV	21 DPV	40 DPV/0 DPI	45 DPV/5 DPI

NV/NC: non-vaccinated/non-challenged; NV/C: non-vaccinated/challenged; RPAdj/C: RNA particle adjuvanted vaccine/challenged; RP/C: RNA particle vaccine/challenged; LE/C: live exposure/challenged; IM: intramuscular; IN: intranasal; DPV: days post vaccination; DPI: days post inoculation.

## Data Availability

The original contributions presented in this study are included in the article. Further inquiries can be directed to the corresponding author(s).

## Abbreviations

The following abbreviations are used in this manuscript:

ABS	Antibiotics
BALF	Bronchioalveolar lavage fluid
BPIV3	Bovine parainfluenza virus 3
BCS	Body condition score
DPV	Days post vaccination
DAB	3,3′-diaminobenzidine
ELISA	Enzyme-linked immunosorbent assay
FFPE	Formalin-fixed, paraffin-embedded
FBS	Fetal bovine serum
HRP	Horseradish peroxidase
IAV-S	Influenza A virus in swine
IHC	Immunohistochemistry
IgA	Immunoglobulin A
IgG	Immunoglobulin G
ISU VDL	Iowa State University Veterinary Diagnostic Laboratory
LE/C	Live exposure, challenged
LRT	Lower respiratory tract
MEM	Minimum essential medium
MHP	*Mycoplasma hyopneumoniae*
NV/C	Non-vaccinated, challenged
NV/NC	Non-vaccinated, non-challenged
PCV2	Porcine circovirus type 2
PIM	Post inoculation medium
PCR	Polymerase chain reaction
PRDC	Porcine respiratory disease complex
PRRSV	Porcine reproductive and respiratory syndrome virus
PPIV1	Porcine parainfluenza virus 1
RP/C	RNA-particle vaccinated, challenged
RPAdj/C	Adjuvanted RNA particle vaccinated, challenged
rtPCR	Real-time polymerase chain reaction
RT-qPCR	Reverse transcription quantitative polymerase chain reaction
SeV	Sendai virus
ST	Swine testicular
TCID50	50% tissue culture infectious dose
TPCK	Tosyl phenylalanyl chloromethyl ketone
TS	Tracheal swab
NT	Nasal turbinate
NS	Nasal swab
SVN	Serum virus neutralization
wvELISA	Whole virus enzyme-linked immunosorbent assay
XIPC	Exogenous internal positive control
